# Flame: an open source framework for model development, hosting, and usage in production environments

**DOI:** 10.1186/s13321-021-00509-z

**Published:** 2021-04-19

**Authors:** Manuel Pastor, José Carlos Gómez-Tamayo, Ferran Sanz

**Affiliations:** Research Programme on Biomedical Informatics (GRIB), Department of Experimental and Health Sciences, Hospital del Mar Medical Research Institute (IMIM), Universitat Pompeu Fabra, Barcelona, Spain

**Keywords:** Modeling framework, Modeling tools, Reproducibility, Model management, Workflow, QSAR, Model integration, Web-interfaces, In-silico toxicology

## Abstract

This article describes Flame, an open source software for building predictive models and supporting their use in production environments. Flame is a web application with a web-based graphic interface, which can be used as a desktop application or installed in a server receiving requests from multiple users. Models can be built starting from any collection of biologically annotated chemical structures since the software supports structural normalization, molecular descriptor calculation, and machine learning model generation using predefined workflows. The model building workflow can be customized from the graphic interface, selecting the type of normalization, molecular descriptors, and machine learning algorithm to be used from a panel of state-of-the-art methods implemented natively. Moreover, Flame implements a mechanism allowing to extend its source code, adding unlimited model customization. Models generated with Flame can be easily exported, facilitating collaborative model development. All models are stored in a model repository supporting model versioning. Models are identified by unique model IDs and include detailed documentation formatted using widely accepted standards. The current version is the result of nearly 3 years of development in collaboration with users from the pharmaceutical industry within the IMI eTRANSAFE project, which aims, among other objectives, to develop high-quality predictive models based on shared legacy data for assessing the safety of drug candidates.

**Supplementary Information:**

The online version contains supplementary material available at 10.1186/s13321-021-00509-z.

## Introduction

In the last years, biomedical data is becoming widely available, thanks to the creation of repositories like PubChem [1] and ChEMBL [2], databases resulting from public–private partnerships like eTOX [3, 4], as well as data policies like FAIR [5], which facilitate the access of existing data to the scientific community.

An interesting way of exploiting this vast amount of data is the development of mathematical models connecting the chemical structure of the substances with their biological properties. Such models are not new. Quantitative Structure–Activity Relationships (QSAR) were first described in the 60 s [6, 7]. QSAR models use regression methods to identify the structural properties linked to quantitative biological properties or to predict these properties for new substances. For biological properties characterized using qualitative descriptions (e.g., positive or negative) conceptually similar approaches can be applied using classifiers. The first QSAR models were developed using small series of congeneric compounds, often synthesized and tested *ad-hoc* for the study. Nowadays, large series of structurally diverse compounds can be easily obtained from public repositories. Pharmaceutical companies can also extract these series from their own internal repositories and use them isolated or combined with compounds from external sources. This fact, combined with recent developments in machine learning (ML) and deep learning (DL) methodologies [8] as well as with the implementation of many of these methods in open source libraries [9], create an ideal scenario for the development of predictive models with biomedical application.

Indeed, the use of ML and DL is becoming very popular in biomedical research. A few remarkable models developed recently have been listed in Table 1 as examples of applications of this methodology, illustrating their usefulness.

**Table 1:** Examples of ML/DL applications in biomedical research

ML application	Brief explanation	References
Drug discovery	Identification of new bioactive compounds	[8–10]
Toxicity prediction	Identify hazardous substances	[11–13]
Precision medicine	Personalize medical treatment to patient idiosyncrasy	[14]
Imaging diagnostics	Identification of abnormalities from imaging	[15, 16]

More and more, the models obtained by the application of ML are seen as valuable business assets. Accurate and appropriately shared models can bring a number of benefits if we are able to make effective use of existing expertise [17]. However, the true capability of a model for solving real-world problems critically depends on aspects related to model implementation, as the following.

### Reproducibility

Models must produce the same results when used at different sites or times. This simple, basic requirement is difficult to meet if (i) the training data is not available and distinctively identified or (ii) the algorithms used are not documented with enough detail, or if it is not possible to use exactly the same software (same version and same platform). The fast evolution of computational tools (both hardware and software) makes it difficult to preserve a model for some time. This topic has been discussed by various authors, proposing diverse solutions for mitigating this problem like the use of appropriate standards for QSAR data interchanges [18] or a workflow for implementing published QSAR models and recommendations to modelers [19].

### Accessibility

Models are digital assets to which the FAIR accessibility principle can also be applied [5, 20]. Ideally, access to existing models should be facilitated, particularly for models developed in academic environments. In practice, there are barriers related to the intellectual property of the tools required to generate the predictions. This can apply to commercial applications used to generate 3D structures or molecular descriptors or even the modeling software itself. For this reason, the use of open source alternatives should be prioritized.

Not all accessibility barriers are related to intellectual property issues, and models should be implemented in a way that allows their use in different operative systems (e.g., Windows, Linux, iOS) and platforms (e.g., implemented as a desktop application or as REST [21] web services in centralized servers). This is particularly true in corporative environments, where company restrictive policies about OS or platforms could hinder access to useful models. Also, and not less important, is to facilitate the model use for non-experts by providing a friendly end-user interface.

### Model management

A good model is a valuable asset for an organization, and as such, it should be managed using appropriate governance principles [17]. One of the first steps is to identify and store the model appropriately. This facilitates common tasks like knowing which model was used to generate some prediction or retrieving a certain model cited in a report. This task is hampered by the fact that models are not static entities. Models evolve as the software they use is updated or as the training series is enriched with new compounds for covering a broader chemical space. Consequently, models often have many versions that must be properly identified and stored as well, recording all the changes in the training series and the modeling software.

A separate task is to document the models. Models can be documented with different levels of detail for different purposes [22]. As a minimum, every model must be accompanied by documents allowing to reproduce the algorithm completely and to understand and interpret the prediction results. The documentation can be used for other purposes, like demonstrating to regulatory bodies the quality of the prediction for replacing experimental tests [23]. Therefore, we recommend a layered documentation structure, including basic mandatory information and more detailed optional layers.

### Reporting

For the model developer, the meaning of a model prediction result is obvious; the model estimates the biological annotations present in the training series. However, users not involved in the process of model building lack this context. This often creates confusion and difficulties for users to interpret the model’s prediction, particularly when the model produces a numerical outcome. For this reason, as a minimum, model results must explicitly include the units in which they are expressed, a brief, concise explanation of how these results must be interpreted, and the level of confidence within which the prediction values must be clearly declared [22].

Every prediction has a certain uncertainty associated as a consequence of the errors present in the training series annotations, as well as the limitations of the model predictivity. For this reason, prediction results must be accompanied by a quantitative estimation of the individual prediction error. This estimation cannot be generic, based solely on the error observed for the compounds in the training series. It must also consider how far the query compound is away from the model applicability domain.

In the last decades, several solutions have been proposed for supporting the access to existing QSAR models or the development of new ones, overcoming the issues described above. One of the first was a Polynomial Neural Network published on-line in 1999 [24]. In a recent review [25], these efforts were classified under four categories; research group-centric model collections, model collections from (Q)SAR oriented projects, (Q)SAR models in integrated modeling environments, and (Q)SAR model repositories. In the present article, we introduce Flame, a new modeling framework for facilitating the development, hosting, and use of predictive models in production environments. When comparing with existing resources, Flame belongs to the category of integrated modeling environments mentioned above. In the “Implementation” and “Results” sections, apart from describing its features, we compare Flame with other similar tools, highlighting the differential characteristics which make it highly valuable for certain QSAR modeling applications.

Flame was developed in the context of project eTRANSAFE (IMI2 Joint Undertaking under Grant Agreement No. 777365), producing integrative data infrastructures and innovative computational methods to improve the feasibility and reliability of translational safety assessment during the drug development process. For this reason, Flame was originally designed to host predictive models for drug safety endpoints, even if it can be used with other applications in biomedical research.

## Implementation

**Fig. 1:**
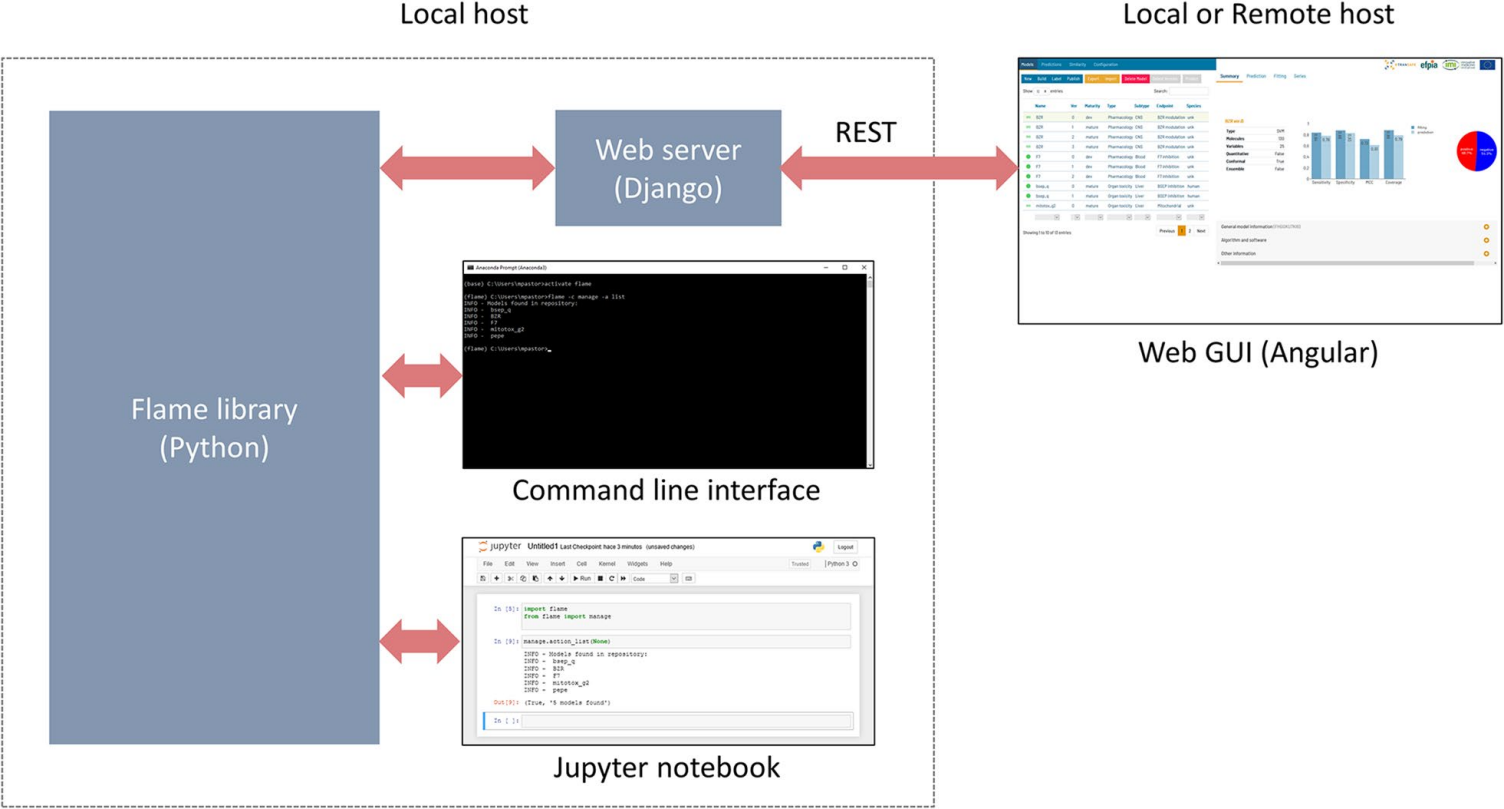
Flame architecture

The Flame architecture is illustrated in Fig. 1. It consists of a Python library (the Flame backend), which can be used from a terminal with a command-line interface, called from a Jupyter notebook [26], or scripts written shell languages (bash, bat, etc.). It also implements a web server (written in Django [27]) offering the library features as REST services [21] and a complete web interface (written in Angular [28]) providing a rich graphic user interface (GUI).

The GUI can be executed locally as a desktop application, starting the web server in the same computer running the Flame backend. It is also possible to run the Flame backend in a server and access the REST services from a remote client, thus allowing to run Flame as a departmental or global prediction service in corporate environments. This architecture differentiates Flame from other integrated modeling environments operating exclusively as web-services (e.g., CHEMBENCH [29], OCHEM [30]). The possibility of executing the software locally, either as a desktop app or in a local server, is a must when the data used for model training or prediction is confidential, and the company policies disallow to send it over the Internet.

The Flame backend and the optional flame web server make use of Conda [31] to define the libraries required, facilitate their automatic installation in a private environment. Conda also allows defining the acceptable library versions to avoid incompatibilities. Flame can be installed and used in Linux, Windows, and iOS operative systems.

**Fig. 2:**
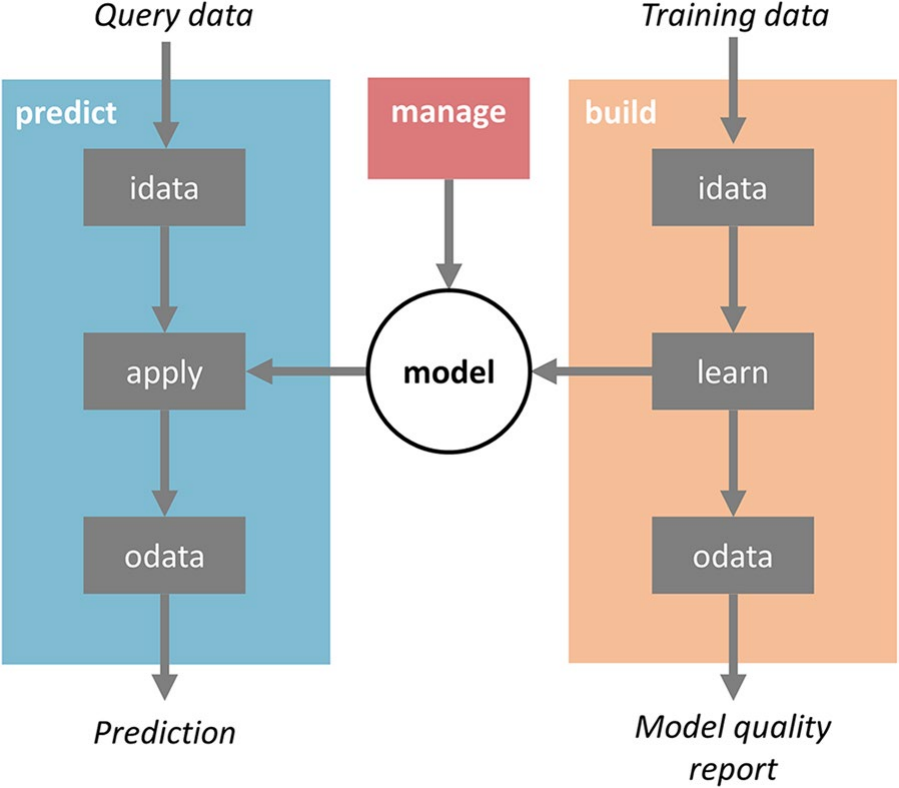
Overview of the main workflows implemented natively in Flame; predict and build. Boxes represent Python classes carrying out specific workflow tasks. As can be seen, some objects (*idata*, *odata*) are reused in both workflows, guaranteeing that the same code is used in model building and prediction

The code was written using Object Oriented Programming (OOP) as a Python library. The main classes (see Table 2 and Fig. 2) can be classified as low-level or high-level. Low-level classes carry out simple tasks while the high-level classes execute model building and model prediction workflows using the low-level classes. For example, the default model building workflow implemented in high-level class *build* (Fig. 2) starts from a training series of annotated chemical structures. It uses class *idata* to import their chemical structures, normalize them, extract the biological annotations and generate molecular descriptors which are stored in a numerical matrix. The molecular descriptors and the annotations are sent to the class *learn*, which normalizes the numerical values and builds models using machine learning (ML) tools like Random Forest (RF). This model is stored in a machine-readable format (as a pickle serialized version of the scikit-learn *estimator object* [9, 32]) suitable to predict the biological properties of novel compounds. Finally, the class *odata* is used to format the results and produce suitable output. The default prediction workflow (Fig. 2) uses exactly the same low-level *idata* class to import and pre-process the structures. This workflow design has the advantage of guaranteeing that the predictions use exactly the same code used for model building, for equivalent tasks, thus producing consistent results. Then, the low-level class *apply* retrieves the *estimator* saved previously during the model building process for computing the prediction results, which are formatted by the *odata* class to generates suitable output.

**Table 2:** Main Python classes used in Flame

Type	Class	Functionality	Input	Output
High-level	Build	Generates a model	Training series	Model
	Predict	Uses an existing model to generate a prediction for a query compound	Query compound	Prediction
	Manage	Handles (create, delete, export and import) models in the repository	–	–
Low-level	Idata	Processes chemical structures to obtain molecular descriptors as an X matrix and annotations as a Y matrix (when provided)	Chemical structures	X (Y) numerical matrices
	Learn	Generates a model from the X and Y numerical matrices	X and Y numerical matrices	Model
	Apply	Uses an existing model to generate a prediction from an X matrix	X numerical matrix	Prediction
	odata	Formats results as human-readable output or computational formats suitable for the GUI	Results	Formatted results

Most of the building and prediction workflow steps are configurable. For example, we can select the structure normalization algorithms or the molecular descriptors calculation method. Also, the methods themselves can be configured by adjusting their internal parameters. In Flame, the methods used to build a model and their configurable parameters are defined in a single parameter file (parameters.yml), which can be seen as the model blueprint. This file is stored in a folder, together with the original training series and the *estimator* generated by *build*. This folder contains a complete and comprehensive definition of how a model has been built. The model repository is a user-defined path in the computer filesystem where all these folders are stored. Flame can work simultaneously with diverse model repositories located in local or remote filesystems, a convenient feature to maintain separate model collections per project or user.

Flame models are used to predict the biological properties of new compounds using the *predict* workflow (see Fig. 2). Since models are folders, they can be saved, compressed, backed-up, or transmitted between Flame instances installed in different computers. In any of these cases, Flame guarantees that the predictions are reproducible. In this sense, Flame models can be seen as self-contained prediction engines. Flame provides commands to export and import models as a single binary file, consisting in the compressed version of the model folder. On import, the version of the libraries used to generate the model is checked to guarantee full compatibility and reproducibility.

**Fig. 3:**
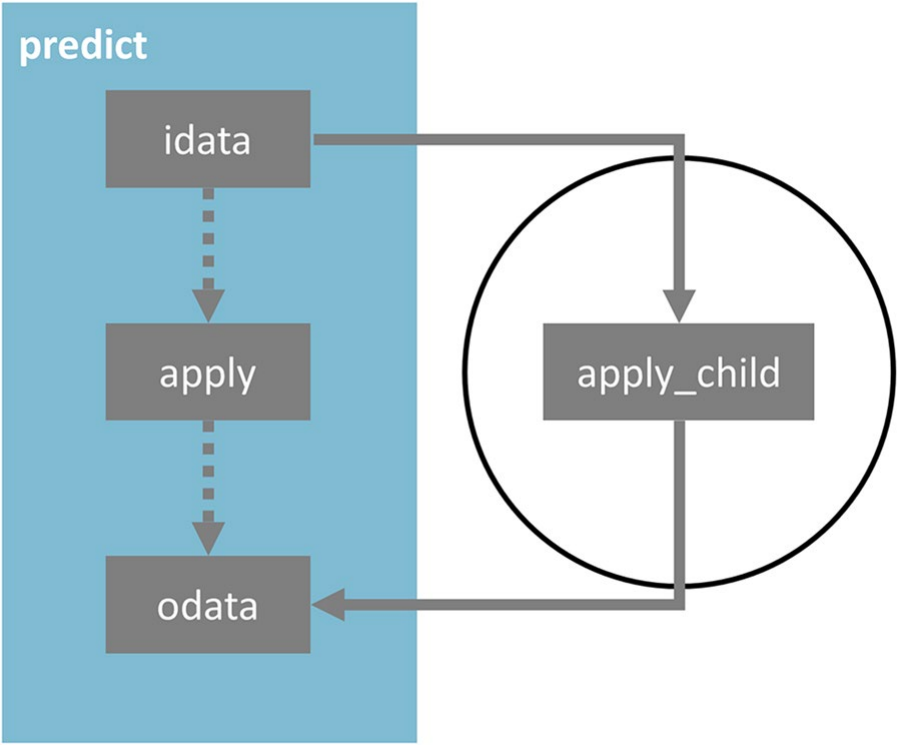
OOP method overriding. Models incorporate children instances of the main low-level classes (see Table 2). By default, the children are empty, and the parent class code is run, but advanced users can edit the code and override the parent class methods to customize the workflow

The use of the parameter file described above offers limited customization since the user can select only among the algorithms and methods implemented natively in Flame. To overcome this limitation, the model workflows do not call the low-level classes directly but use a derived class stored locally within the model folder (see Fig. 3). These derived or child classes inherit all of the parent class properties, and in simple models this mechanism is the exact equivalent to calling the Flame classes directly. However, the child class methods can be overridden, allowing unlimited model customization. For example, advanced users can insert code calling external tools to generate molecular descriptors, include extra steps in the model building or prediction workflow or adapt the output to generate customized reports. Since these changes are written in the child class instance, stored locally within the model folder, they do not affect other models. Moreover, these changes are preserved when the model is saved or exported. This possibility to embed custom code in the building and prediction workflows differentiates Flame from other integrated environments, either on-line or downloadable. To the best of our knowledge, it is only present in eTOXlab [33], a modeling framework developed in our group some time ago.

## Results

### Model building features

Flame can build predictive models starting from a single file in SDFile format containing the structures and the biological properties of a training series. The default model building workflow takes care of reading the structures, normalizing them, extracting the annotations, generating molecular descriptors, scaling their values and building a machine-learning model that is saved in a format suitable for predicting new compounds’ properties.

**Fig. 4:**
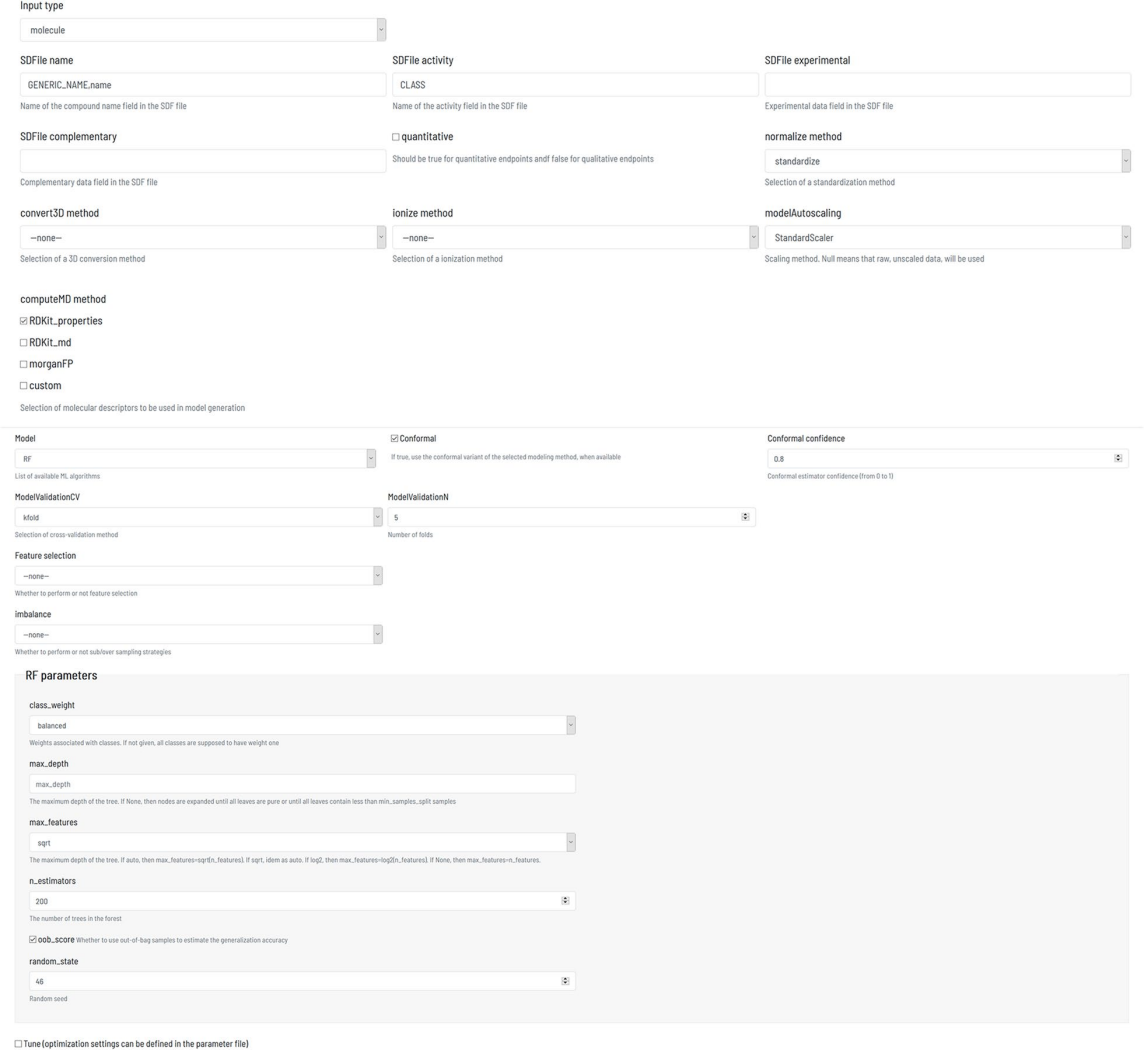
Dialogue used to define the model building workflow methods and parameters (simplified)

Flame provides defaults for methods and parameters, but the user can customize them, either editing the parameter file parameters.yml when using Flame in command line mode or using the model building dialogue (Fig. 4) when using the Flame GUI.

Table 3 describes the methods implemented natively in Flame. All of them make use of open source libraries. The choice of models can be easily extended to include commercial products or external tools, using the code overriding technique described in “Implementation” section.

**Table 3:** Overview of the main modeling methods and tools implemented natively in Flame

Modeling task	Method	Source
Structure normalization	Standardiser	[34]
	ChEMBL pipeline	[35, 36]
Molecular descriptors calculation	RDKit properties	[37]
	RDKit md	[37]
	RDKit Morgan fingerprints	[37]
Scaling	Raw	–
	Autoscaling	[9]
Machine learning	RF	[9, 38]
	SVM	[9, 39]
	PLS	[9, 40]
	XGBOOST	[41]
	Conformal regression	[42, 43]

Typically, models are built starting from a collection of annotated chemical structures, but Flame can also use as input a tab-separated (TSV) table with pre-calculated molecular descriptors and annotations. Another option, rarely found in other modeling frameworks (but present in OCHEM [30]), is the possibility to use as input the prediction results of other models present in the repository. This option, called in Flame “model ensemble”, is interesting for integrating the results of multiple models. For qualitative models, multiple results can be summarized using majority voting. The prediction results of an ensemble of quantitative models can be summarized using their means or medians. Regressors and classifiers can also be trained with the model ensemble, using it as a sort of “molecular descriptors”, to generate a smarter result combination and obtain better predictions. When the ensemble models estimate the individual prediction error, this information is considered by Flame, using appropriate probabilistic methods, to generate an estimation of the final prediction error. The description of these algorithms is beyond the present work scope and will be published in a separate article.

The last step of model building workflows is estimating the model quality using cross-validation. Flame presents information about the model goodness of fit, predictive quality, and some statistical information of the training series (e.g., value distribution). Since Flame can use diverse ML methods, we tried to generate comparable model quality indexes to facilitate the selection of the best methods and parameters. The values shown are summarized in Table 4 for qualitative and quantitative endpoints.

**Table 4:** Model quality parameters shown in the Flame GUI

Endpoint type	Parameter	Definition
Qualitative	Sensitivity (fitting and prediction)	$\frac{TP}{{TP + FN}}$
	Specificity (fitting and prediction)	$\frac{TN}{{TN + FP}}$
	MCC (fitting and prediction)	$\frac{{\left( {TP*TN} \right) - \left( {FP*FN} \right)}}{{\sqrt {\left( {TP + FP} \right)\left( {TP + FN} \right)\left( {TN + FP} \right)\left( {TN + FN} \right)} }}$
Quantitative	SDEC (fitting)	$\sqrt {\frac{{\sum \left( {Y_{exp} - Y_{pred} } \right)^{2} }}{n}}$
	SDEP (prediction)	$\sqrt {\frac{{\sum \left( {Y_{exp} - Y_{pred} } \right)^{2} }}{n}}$
	r^2^ (fitting)	$\frac{{\sum \left( {Y_{exp} - Y_{pred} } \right)^{2} }}{{\sum \sqrt {\left( {Y_{mean} - Y_{pred} } \right)} }}$
	q^2^ (prediction)	$\frac{{\sum \left( {Y_{exp} - Y_{pred} } \right)^{2} }}{{\sum \sqrt {\left( {Y_{mean} - Y_{pred} } \right)} }}$
Conformal models	Conformal coverage	$\frac{Samples\;inside\;confidence\;boundaries}{{Total\;number\;of\;samples}}$
	Conformal accuracy	$\frac{Samples\;predicted\;correctly}{{Total\;number\;of\;samples}}$
	Mean interval (only quantitative)	$\frac{{\sum \left| {Y_{max} - Y_{min} } \right|}}{n}$

**Fig. 5:**
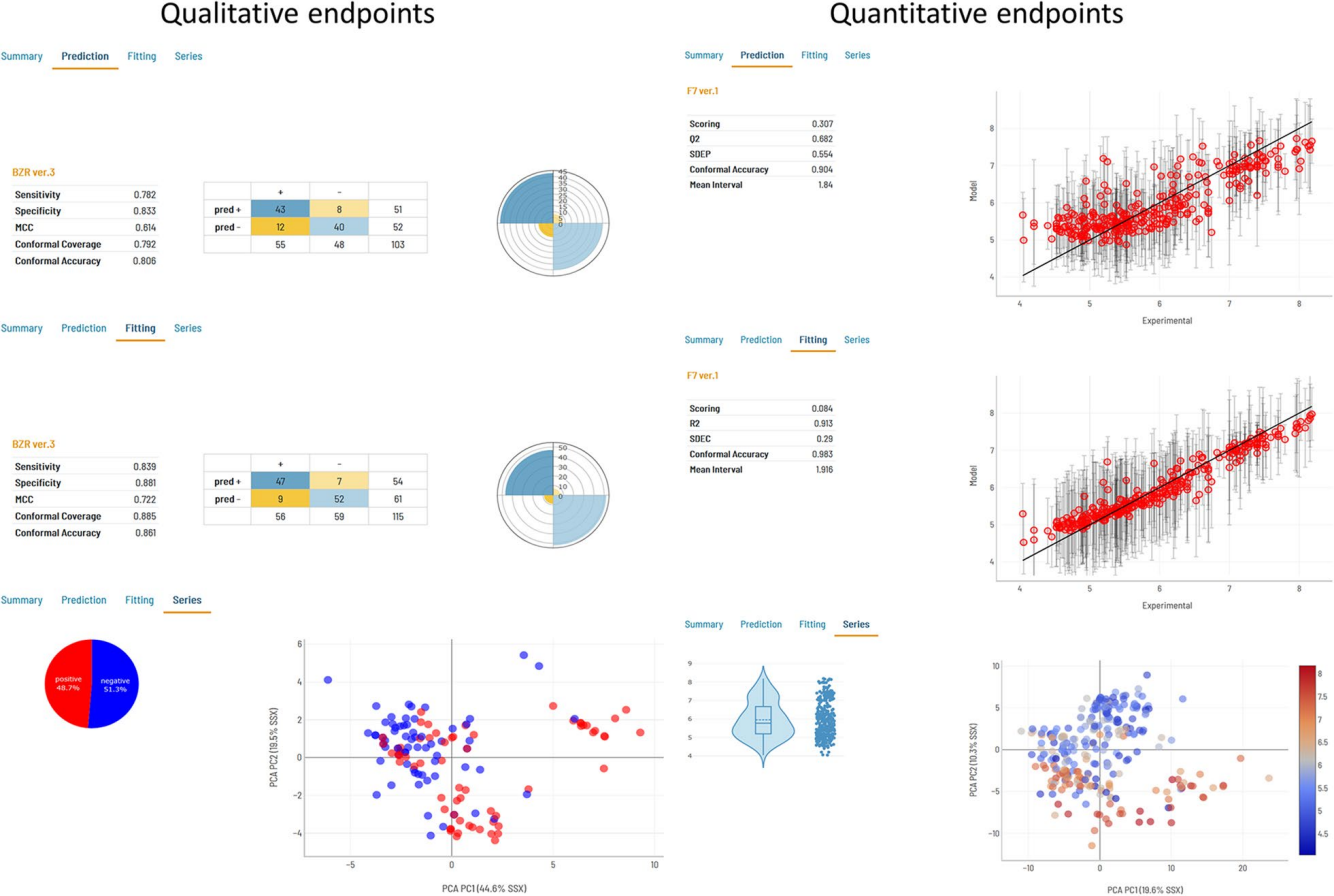
Model output for qualitative (left) and quantitative (right) endpoints

The Flame GUI provides additional information oriented to diagnose the quality of the model and the training series, as shown in Fig. 5. For qualitative endpoints (left side of Fig. 5), the confusion matrix is shown as a 2 × 2 matrix. A radar plot is also used to represent, in the radius of its four sections, the relative size of the true positive, true negative, false positive, and false negative results. This information is shown separately for the model fitting and prediction, the latter being calculated using cross-validation methods selected by the user (default to five k-fold). Besides, Flame displays a scatterplot of the training series using the two first Principal Components (PCs) obtained by running a Principal Component Analysis (PCA) with the calculated molecular descriptors. Objects (compounds) are colored red or blue according to their biological annotations (positive or negative, respectively). The positive and negative ratio of substances in the training series is depicted using a pie chart.

For quantitative endpoints (right side of Fig. 5), apart from the parameters mentioned in Table 4, the interface shows scatterplots of fitted/predicted values versus the experimental annotations. For conformal models, the confidence interval for the defined confidence level is also shown. Flame displays a scatterplot of the training series in a separate tab, like the one shown for qualitative endpoints. However, in this case, the substances are colored using the continuous scale included in the plot. The distribution of the annotation values is shown using a violin-type plot, which offers valuable information to diagnose a skewed value distribution or the presence of outliers. All the graphics representing the training series are interactive, and hovering the mouse cursor over the dots allows to display the 2D structure of the compounds they represent.

The model quality reports described above are persistent. All this information is stored within the model folder and can be retrieved and shown in subsequent work sessions.

### Model predictions

Models stored in the repository can be used to predict the biological properties of a compound entering an SDFile with its structure or sketching it in the included molecular editor. The prediction workflow will then apply to this structure the same pretreatment, molecular descriptors calculation, and x-matrix scaling used for the training series, using exactly the same source code, thus guaranteeing the maximum consistency. The molecular descriptors obtained are projected using the stored estimator to obtain the prediction results. Prediction results can be qualitative or quantitative, depending on the nature of the training series annotations. Models built using conformal regression [43] generate additional information about the prediction uncertainty. For quantitative endpoints, they provide a confidence interval, while for qualitative (binary) endpoints, the prediction result can be “uncertain”, meaning that the model cannot ascertain if the result is positive or negative. In either case, the model reports the prediction uncertainty at a probability (the CI confidence level or the probability that the result is correct, respectively) defined by the user.

Models are watermarked using a unique ID (a random string of ten ASCII uppercase chars) generated during the model building process. This ID is useful to guarantee the model identity, even when different models are assigned the same name or exported to different Flame instances. Furthermore, when a model is used for prediction, its unique model ID is stored together with the prediction results. Predictions stored in the prediction repository keep record of the model version used to generate it, guaranteeing complete traceability.

As stated in the introduction, prediction results are often difficult to understand and interpret by users not involved in the model building. For this reason, the Flame GUI presents the prediction results in various formats, decorated with extra information aiming to facilitate the result interpretation and its use for decision making.

**Fig. 6:**
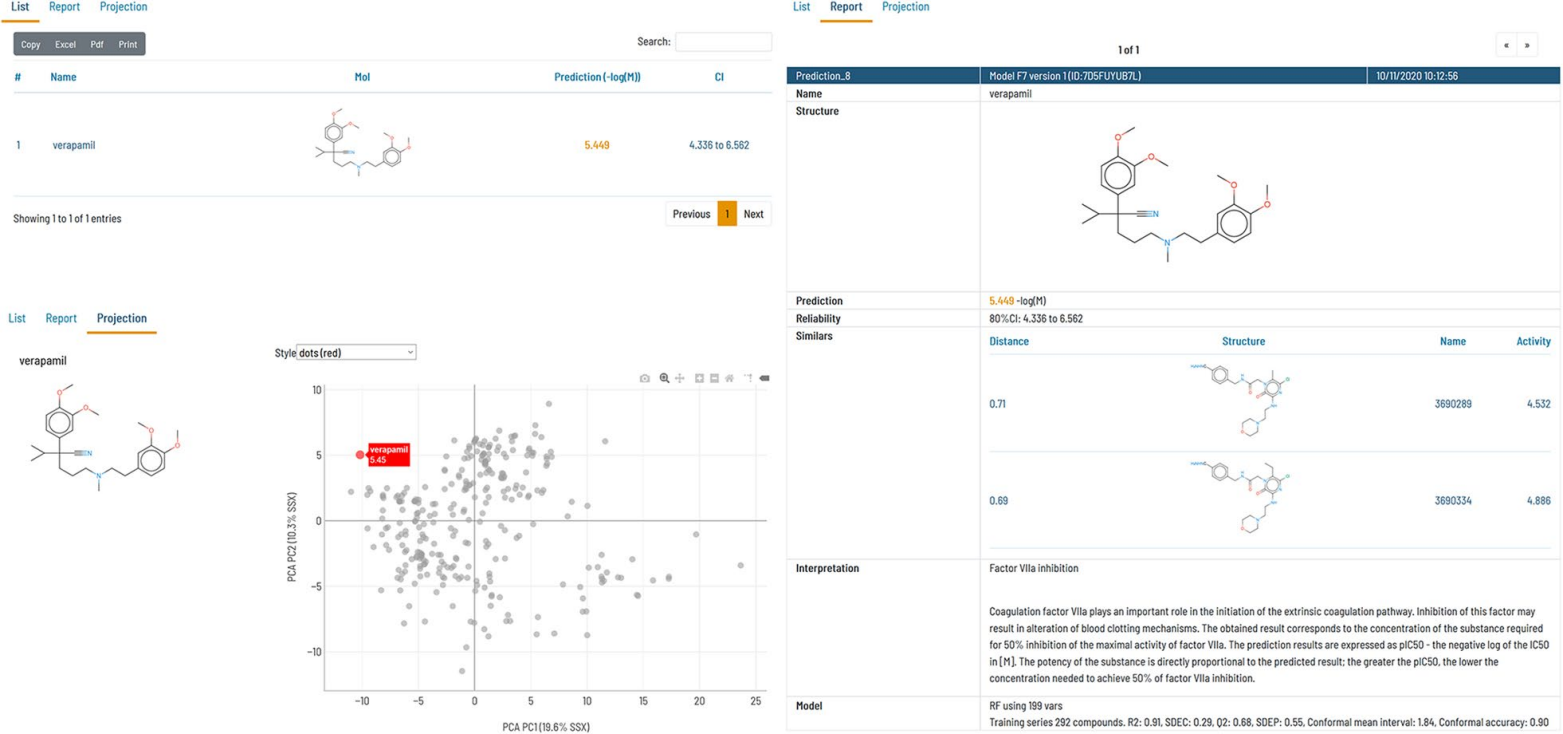
Representation of the model prediction results in the Flame GUI

As shown in Fig. 6, results are displayed in three alternative views. First, they are presented as a list, including for every predicted compound its name, 2D structure, prediction result, and uncertainty information when available. This list is paged, searchable, and can be ordered by column values. It can also be exported to Excel or PDF formats, printed, or copied to the clipboard. Clicking in any of the list items displays a more detailed report for a single compound. Reports show the compound name, structure, and prediction result as well as complementary information about how to interpret the result (extracted from the model documentation). Besides, a list of the closest compounds in the training series, labeled with their biological annotations, is also shown. For obtaining this list, the similarity is computed using the same molecular descriptors used for building the model.

**Fig. 7:**
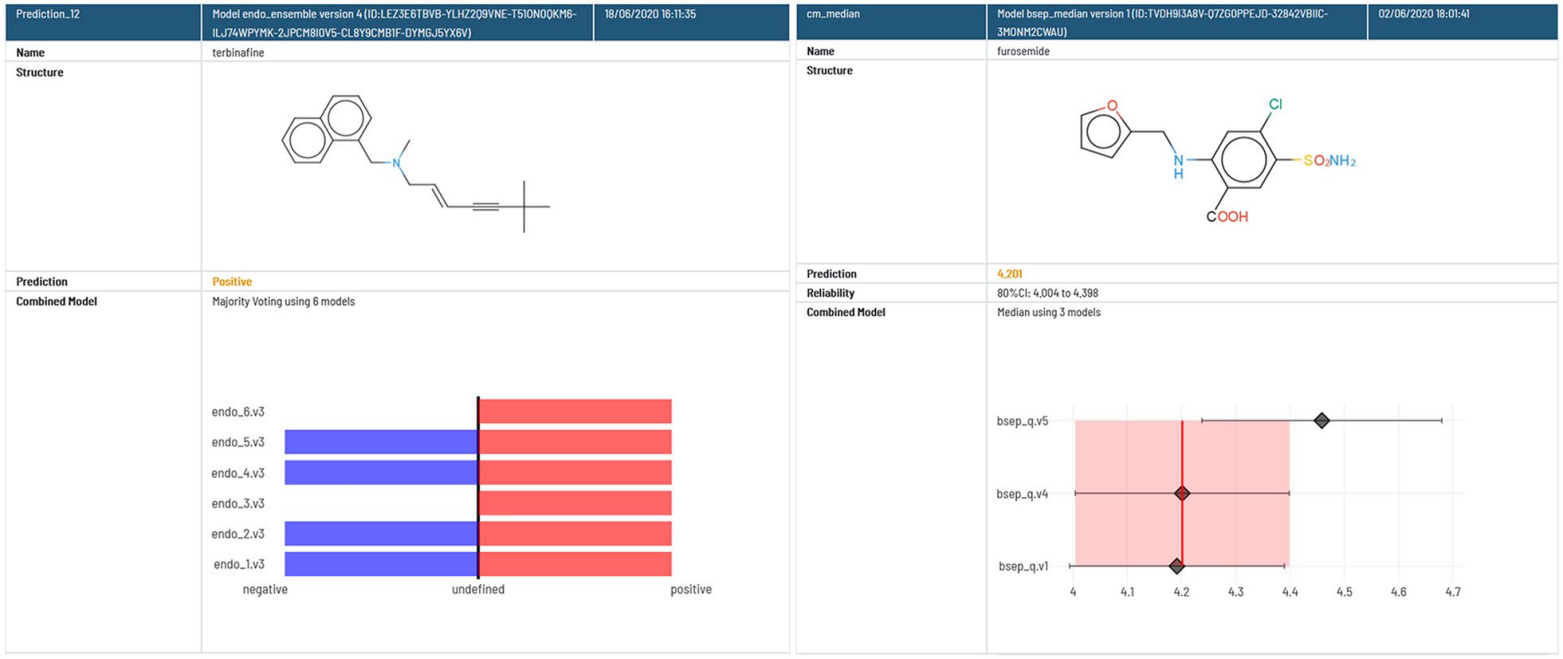
Visualization of prediction results obtained with ensemble models for qualitative (left) and quantitative (right) endpoints

When an ensemble of models is used for prediction, the prediction report shows the individual result of the low-level models and the combined result (Fig. 7). For conformal binary classifiers (left of Fig. 7), the graphic shows the low-level model prediction results, indicating if the query compound belongs to class 0 (negative), class 1 (positive), both of them (inconclusive type I) or neither (inconclusive type II). For conformal quantitative models (right of Fig. 7), the predictions are shown with the corresponding confidence intervals.

Finally, the prediction results are also projected on the training series PCA scores scatterplot, generated as explained in the previous section (Fig. 5). The aim of this representation is to show whether the predicted compound belongs to a region of the chemical space well represented by the training series or if it belongs to a less populated region. In this representation, the training series compounds can be displayed as grey dots or colored by the biological annotation. The predicted compound can be displayed as green circles with the compound names, as red dots, or as dots colored by the compound distance to model (DModX, see [44]). A high DModX value indicates that the predicted compound has original features not present in the training series, which can be detrimental to the prediction quality.

Finally, it should be mentioned that the predictions are stored in a persistent prediction repository, and therefore, it is possible to revisit previous predictions until they are actively removed by the user.

### Model management

Once a model is built, it is stored in a separate folder of the model repository. This folder can contain multiple versions of the model. As a minimum, there is a *dev* version that is used for model development and is overwritten every time the model is re-built. Precisely for this reason, the *dev* version cannot be used for prediction. Model versions that the model developer considers worth storing should be published to generate version 1, 2, etc.

**Fig. 8:**
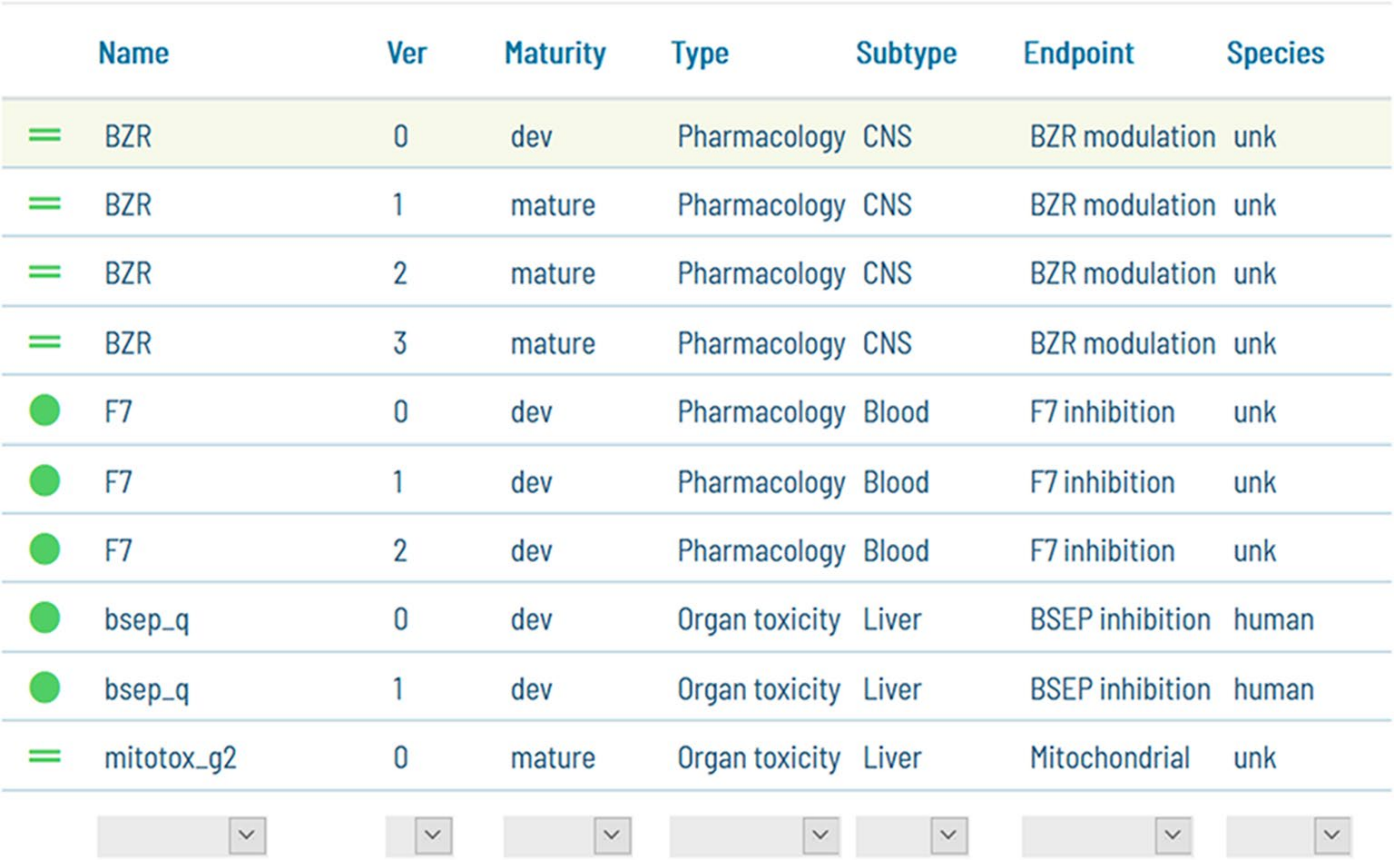
The models present in the model repository are shown in the GUI as a model list. Items can be sorted, browsed, and searched by text terms. Models include user-defined labels which can be used to filter the list content

The main GUI window shows a list (Fig. 8) where models can be browsed and selected. Every model is identified with a name and version and labeled by Maturity, Type, Subtype, Endpoint, and Species. The labels are defined by the end-user and can be used to filter the models shown, making it easier to find models for a particular endpoint, species, or organ.

The command mode interface and the GUI provide model management commands for creating new models, publishing a model version, deleting a whole model tree with all the versions or any single model version.

Models can be exported using a command that produces a compressed version of the whole model folder. This file can be easily stored, backed-up, or sent in electronic formats (e.g., as an e-mail attachment). Once imported in any Flame instance, the model is copied to the model repository and becomes fully functional. During the importing step, the versions of the software libraries used for generating the models are checked, and in case of version mismatches, a warning message is shown.

### Model documentation

Flame models are documented using a template based on the QMRF [45], taking advantage of our previous experience in model documentation [22]. When the model is built, Flame automatically completes in this template the fields describing the modeling methodology and quality. This half-completed document should be edited by the modeler, using the GUI or editing a documentation file in *yaml* format using a text editor and importing it into the model. In either case, the model documentation is stored in the model folder and is included when the model is exported or published.

The model documentation has been split into three sections: General Model information, Algorithms, and Other information. The first and third sections should be completed by the modeler, while Flame automatically completes most of the second section. The Additional file 1 contains an example of a human-readable file in *yaml* format, suitable for being imported into a Flame model, with all the items included in these three sections. The Additional file 2 contains a PDF file showing how the model documentation is presented to the user in the Flame GUI.

### Performance

In a typical modeling workflow, the same code (structure normalization, molecular descriptors calculation) is run for every compound in the input series, both for training series and prediction series. Therefore, the computation can be speed-up by splitting the series into *n* sub-series and assigning them to different computation threads, which are run in different CPUs. Flame can run in parallel the workflow tasks related to the calculation of the molecular descriptors, obtaining nearly linear speed-up. Another time-consuming step is model building and validation. By default, Flame applies the parallel processing implemented in the ML libraries (e.g., scikit-learn implements parallel processing in cross-validation and grid-search, while XGBoost uses it in the model building and validation). The use of GPUs is under development. A special Flame version supporting GPUs is planned to be released in the future, facilitating the efficient use of deep learning within the framework.

Additionally, during model development, it is common to rebuild the model repeatedly using diverse machine learning settings to optimize them. To speed up this process, Flame stores intermediate results of the calculation (e.g., the molecular descriptors matrix), thus saving the work of re-computing them in every cycle.

Flame has been used to develop models using series of very different size and characteristics. To give an idea of Flame performance and limitations we have included Table 5 with some benchmarking results.

**Table 5:** Computation time for series of diverse size

Series	Original series size	Compounds removed^a^	Final series size	Time (s)^b^
A	2685	11	2674	20 s
B	5832	0	5832	32 s
C	126,368	114	126,254	600 s (10 min)
D	480,000	249	479,751	2160 s (36 min)

Computation tasks involving structure normalization, computation of RDKit descriptors, generation of a XGBoost model, and validation using fivefold validation (for series A and B) and twofold validation (for series C and D).

^a^Compounds removed from the computation because RDKit was unable to compute molecular descriptors

^b^Wall clock times, in a desktop PC with Windows 10 professional 64 bits, 32b Gb RAM and an AMD Ryzen 5 3600(6 cores) CPU

### Error handling

Any modeling software aiming to solve real-life problems should know how to deal with errors present in the input files. These errors can stop the modeling workflow for many reasons: input molecules can have a wrong structure, contain metals, counterions or water molecules. The model building can also fail when the annotations are not correct. For this reason, a lot of effort was devoted to implementing appropriate error handling methods in Flame, able to identify and remove automatically molecules that cannot be processed and producing informative error reporting both in the GUI and the console. As an example of Flame robustness, the D series in Table 5 contains 480,000 structures extracted directly from ChEMBL, with no curation. Flame was able to process the series removing automatically 249 structures (0.05%), for which RDKit was not able to generate molecular descriptors. Modelers know that there are many potential sources of error, and Flame does not claim to be able to handle all error types. However, years of development and use by different modeling teams established Flame as a rather robust software.

### Comparison with other integrated modeling environments

As mentioned in the introduction, many tools for supporting the development of QSAR models are available. A comparison of Flame with a representative sample of related software would be helpful to highlight its advantages. This comparison is focused on software that can be installed locally, discarding purely on-line tools like CHEMBENCH [29] or OCHEM [30]. In many cases, these are excellent options, but in situations where the models should be trained with confidential data or used to predict confidential data, they are not usable. Indeed, Flame was developed specifically for supporting modeling activities in the project eTRANSAFE, aiming to develop predictive models for the pharmaceutical industry using their confidential data. Another selection criterion is software accessibility. For example, OpenMolGRID [46] was one of the first integrated modeling environments, but it is not accessible anymore. Also related to the accessibility, commercial software, and software requiring registration or special agreements (e.g., QSARIN-chems [47] and ChemProp [48]) is excluded from the comparison, focusing our attention on open source freely accessible tools. After applying these criteria, we have selected the tools listed in Table 6 as a representative sample of the state-of-the-art, which does not intend to be exhaustive.

**Table 6:** Locally installable software usable as an integrated modeling environment

Name	Version	License	Platform	Language	Model prediction	Model building
eTOXlab [33]	0.9.6	GNU GPL-3	Any	Python/VM	Yes	Yes
VEGA-QSAR [49]	1.1.5.47	GNU GPL-3	Any	Java	Yes	No
EPI-suite [50]	4.11	Copyright EPA, free of use	Windows		Yes	No
Kausar Automated-framework [51]	na	na	Any	KNIME	Yes	Yes
ToxTree [52]	3.1.0	GNU GPL-2	Any	Java	Yes	No
OECD QSAR ToolBox [53]	4.4.1	requires registration	Windows		Yes	No

The first tool listed, eTOXlab [33] was developed in our group, and part of its conceptual design was reused in Flame. It is an integrated modeling framework developed in Python and distributed as a source code or pre-installed in a virtual machine. It can develop new models starting from an annotated SDFile, store models in a repository, and use them for prediction. As in Flame, models can include children of the source coded classes for model customization. However, eTOXlab offers limited features, and, for example, it cannot be used as a web service, has a more limited panel of methods, and its GUI is primitive. VEGA-QSAR [49] and EPI-suite [50] are prediction-oriented tools containing a collection of very useful models, but they lack the Flame ability to develop new models. The Kausar Automated framework for QSAR model building [51] is a fully featured collection of KNIME workflows for model development and prediction. However, it is oriented mainly to model developers and lacks an interface that makes it suitable for end-users. Moreover, KNIME is not open source, hampering its installation in non-academic environments.

The two remaining tools in the table are special cases. ToxTree [52] is a tool for the estimation of Toxic Hazard using only the decision tree approach. The OECD QSAR ToolBox [53], in spite of its name, is not specifically aimed to develop or apply QSAR models. Its scope is broader, oriented to obtain chemical hazard assessments by retrieving experimental data from internal databases, simulating metabolism, and profiling the chemical properties of chemicals. This information is then used for read-across, finding structurally and mechanistically defined analogs and chemical categories.

## Discussion

The development of Flame was justified by the need for an integrated modeling framework in the eTRANSAFE project, meeting its specific needs as well as providing pragmatic solutions to the general requirements of any predictive model listed in the introduction. How Flame addresses these requirements?

### Reproducibility

Models generated and stored in Flame are fully reproducible across Flame instances and can be easily exported and imported, always obtaining the same results. The use of controlled Conda environments and the tagging of the library versions used during the model generation provides reasonable control of the software libraries and versions used. However, Flame cannot avoid the obsolescence of the software and hardware. For medium to long-term model storage, saving images of the whole system using docker or virtual machines is recommended.

### Accessibility

Flame is open source and uses only open source software. It is available in the most popular operative systems (Linux, Windows, and iOS). It can be used as a desktop application with a rich GUI, from the command line, integrated into scripts, in Jupyter notebooks, or as a web service. The GUI was designed for non-expert users, but experienced modelers can customize the models without limitations. Additionally, Windows and Linux installers are distributed on the GitHub page to facilitate its installation by non-expert users. These installers are self-contained, including all the libraries needed to install and run Flame without an Internet connection. This is an essential feature for its installation in corporate environments where security is critical and Internet connection is either blocked or filtered.

### Governance

Flame incorporates advanced model management tools, supporting the whole model development cycle. Models can be developed, improved, and stored in a persistent model repository, where they can be labeled using up to four types of keywords. Models are also thoroughly documented using widely accepted standards and given a unique ID. The documentation is organized in sections using a structure close to the layered approach proposed in the introduction.

### Reporting

Flame predictions are presented to the users in a variety of formats, some of them specifically designed to facilitate the interpretation by non-expert users, providing contextual information about the biological annotations and the result interpretation. Whenever the model allows, the prediction result is presented with information about its uncertainty, using rigorous formalisms (e.g., conformal regression) expressed in formats familiar to experimentalists (confidence intervals).

## Conclusions

We presented Flame, an open source modeling framework that can be used for the easy development of QSAR-like models. The incorporated model building workflow only requires the input of a single annotated SDFile to generate a model, using default options. This workflow can be easily customized to use any of the natively supported methods and a wide variety of method parameters. Moreover, it incorporates mechanisms to implement unlimited customization by using model-linked source code overriding.

Many predictive modeling applications depend critically on addressing implementation issues that hinder the use of models in production environments. Our modeling framework provides reasonable solutions for most of these issues and facilitates a seamless transition from model development to model production with little effort. Models can be easily maintained, stored, exported, and imported, facilitating the collaboration between academic and private institutions.

Flame uses innovative methods to combine models by building models based on the results of other models. This adds unique flexibility for combining multiple models addressing the same endpoint or combining models representing multiple mechanisms contributing to the same endpoint in the toxicological field. Some interesting applications of this model combination tool have been obtained and will be published in due time.

Flame incorporates a rich web-based GUI, facilitating the model building, administration, and use in prediction. Prediction results are presented to the user in various formats, including information like the substances in the training series closer to the predicted compound or projections of the query compounds on the training series chemical space.

Flame has been developed within eTRANSAFE, a large European project involving numerous farmaceutical companies, some of which are testing Flame internally. The feedback obtained in this interaction has been a precious resource for designing a tool that can help drug developers and drug safety experts in their daily work.

The comparison with similar tools, installable locally, was favorable to Flame and highlighted some of its advantages. If the comparison is extended to include on-line services like CHEMBENCH and OCHEM, these platforms outperform Flame in some aspects, even if they lack the advanced model customization offered by locally installable tools.

For all these reasons, Flame can be considered a very useful tool with unique features. As yet, it does not incorporate all the modeling tools available, but we plan to keep enriching their features, incorporating other molecular descriptor generators and machine learning toolkits. In this respect, we plan to expand the Flame user’s community beyond the eTRANSAFE consortium and interest developers that can contribute their code in future versions.

## Supplementary Information

**Additional file 1.** Model documentation exported in human-readable yaml format that can be edited and imported.

**Additional file 2.**  Large figure showing the aspect of the model documentation GUI in Flame. 

## Data Availability

Flame source code is available at GitHub under GNU GLP-3.0 license at the following repositories: https://github.com/phi-grib/flame (backend), https://github.com/phi-grib/flame_API (web server), https://github.com/phi-grib/flameWeb2 (frontend). No dataset was described nor required to support the conclusions of the manuscript. Project name: Flame. Project home page: https://github.com/phi-grib/flame (backend), https://github.com/phi-grib/flame_API (web server), https://github.com/phi-grib/flameWeb2 (frontend). Operating system(s): Platform independent. Tested in Windows, Linux, and iOS. Programming language: Python, Typescript (Angular). Other requirements: Flame uses a Conda environment defining dependencies to other Python libraries. License: GNU GPL-3.0
