# Supplementary material for: Flame: an open source framework for model development, hosting, and usage in production environments
Source: J Cheminform. 2021 Apr 19;13:31. doi: 10.1186/s13321-021-00509-z (PMC8054391; doi:10.1186/s13321-021-00509-z)
Supplement: Supplementary file 2 — Additional file 2. Large figure showing the aspect of the model documentation GUI in Flame. [file 13321_2021_509_MOESM2_ESM.pdf]

General model information (FHGGKU7KI6)

Edit

	Value	Description
ID	BZR	Model identifier
Version	Development 27	Version of the model
Contact	Karolina Kopanska (karolinaweronika.kopanska@upf.edu)	Model identifier
Institution	Universitat Pompeu Fabra (UPF), Research Program on Biomedical Informatics (GRIB) , PharmacoInformatics group (PhI)	Affiliation details
Date	22.07.2020	Date of model development
Endpoint	Allosteric modulation of benzodiazepine receptor	Endpoint modeled
Endpoint units	Class prediction (active or inactive)	Response units to model
Interpretation	Benzodiazepine receptors are allosteric modulatory sites on GABAA receptors. The effect of the benzodiazepine receptor modulation is the potentiation of the neural inhibition that is mediated by gamma-aminobutyric acid (GABA). The BZR model classifies substances as active or inactive, thereby predicting their physiological effect on the receptor. A positive result indicates that the particular substance was classified as BZR modulator.	Interpretation of the model
Dependent variable	Activity class membership	Response variable used to model
Species		Modeled endpoint species
Limits applicability		Applicability limits of the model
Experimental protocol		Description of the followed experimental protocol
Model availability	description — Additional information<br>license — Model license specifications<br>location — Model URL/place	Information about model availability
Data info	format: SDF — Format used (SDF, SMILES, table..)<br>pre-procesing: Mol blocks were computed from SMILES using RDKit.Chem — Details on data processing<br>raw data url: https://pubs.acs.org/doi/suppl/10.1021/ci5001168/suppl_file/ci5001168_si_001.xlsx — URL of the original data<br>test set size — Size of the test set<br>test set url — Test set URL<br>training set size: 130 — Size of the training set<br>training set url — Training set URL	Model data resources

Algorithm and software

Edit

	Value			Description
Algorithm	algorithm	SVM	Type of algorithm (Random Forest, SVM, etc)	Description of the algorithm
	descriptors	RDKit_properties	Name of descriptor package/s used	
	type	QSAR	Type of model (QSAR, RA, etc)	
Software	applicability domain		Applicability domain software, version, description and license.	Description of the used software
	descriptors	RDKit	Software used to generate descriptors, version, description and license	
	model	Flame	Main modelling program, version, description and license.	
Descriptors	descriptors		Descriptors used, list	Information on descriptors used for the model
	final number	25	Final number of descriptors after feature selection	
	initial number		Initial number of descriptors	
	ratio	0.19	Ratio samples/descriptors	
	selection		Final list of descriptors used	
	selection method		Feature selection technique	
Algorithm settings	C	1	na	Algorithm parameters information
	break ties	false	na	
	cache size	200	na	
	class weight		na	
	coef0	0	na	
	decision function shape	ovr	na	
	degree	3	na	
	gamma	auto	na	
	kernel	rbf	na	
	max iter	-1	na	
	probability	true	na	
	random state	46	na	
	shrinking	true	na	
	tol	0.001	na	
	verbose	false	na	
AD method	description	Adding conformal features to the classifier guarantees both, a certain level of confidence and a maximum frequency of errors (misclassifications) which will be committed by the conformal predictor. The given confidence level opposes the error rate which represents the level of uncertainty that must initially be accepted and provided by the user. Conformal accuracy indicates the fraction of correct predictions at the given confidence level. Conformal coverage is the fraction of substances for which valid predictions were generated at the required confidence level.	Description of the applicability domain technique	Applicability domain technique details
	name	conformal prediction	Name of the applicability domain technique	
AD parameters	confidence	0.8	Conformal confidence (from 0 to 1)	Parameters of the applicability domain technique
Goodness of fit statistics	Specificity	0.83	na	Goodness of fit statistics
	MCC	0.61	na	
Internal validation 1	Conformal coverage	0.79	na	Internal validation method and statistics for internal cross-validation
	Conformal accuracy	0.81	na	
Internal validation 2				Additionl validation settings and statistics for internal cross-validation
External validation				External validation statistics
Comments				Additional commnents on model building

Other information

Edit

	Value	Description
Other related models		Related models
Date of QMRF		Date of QMRF, usually is the same as the model creation
Date of QMRF updates		Dates of document updates
QMRF updates		Update of the QMRF
References		Reference(s) to main scientific papers and/or software package
QMRF same models		Availability of another QMRF for exactly the same model
Comment on the endpoint		Additional comments on the modeled endpoint
Endpoint data quality and variability		Text field containing all the information for adequately judge the quality of the experimental data.
Descriptor selection		Detailed information about descriptor selection process
